# Hyperspectral Imaging Using a Convolutional Neural Network with Transformer for the Soluble Solid Content and pH Prediction of Cherry Tomatoes

**DOI:** 10.3390/foods13020251

**Published:** 2024-01-12

**Authors:** Hengnian Qi, Hongyang Li, Liping Chen, Fengnong Chen, Jiahao Luo, Chu Zhang

**Affiliations:** 1 School of Information Engineering, Huzhou University, Huzhou 313000, China; 2 Huzhou Agricultural Science and Technology Development Center, Huzhou 313000, China; 3 School of Artificial Intelligence, Hangzhou Dianzi University, Hangzhou 310018, China

**Keywords:** tomato quality, wavelength weight visualization, deep learning regression, non-destructive testing

## Abstract

Cherry tomatoes are cultivated worldwide and favored by consumers of different ages. The soluble solid content (SSC) and pH are two of the most important quality attributes of cherry tomatoes. The rapid and non-destructive measurement of the SSC and pH of cherry tomatoes is of great significance to their production and consumption. In this research, hyperspectral imaging combined with a convolutional neural network with Transformer (CNN-Transformer) was utilized to analyze the SSC and pH of cherry tomatoes. Conventional machine learning and deep learning models were established for the determination of the SSC and pH. The findings demonstrated that CNN-Transformer yielded outstanding results in predicting the SSC, with the coefficient of determination of calibration (R^2^_C_), validation (R^2^_V_), and prediction (R^2^_P_) for the SSC being 0.83, 0.87, and 0.83, respectively. Relatively worse results were obtained for the pH value prediction, with R^2^_C_, R^2^_V_, and R^2^_P_ values of 0.74, 0.68, and 0.60, respectively. Furthermore, the visualization of the CNN-Transformer model revealed the wavelength weight distributions, indicating that the 1380–1650 nm range served as the characteristic band for the SSC, while the spectral range at 945–1280 nm was the characteristic band for pH. In conclusion, integrating spectral information features with the attention mechanism of Transformer through a convolutional neural network can enhance the accuracy of predicting the SSC and pH for cherry tomatoes.

## 1. Introduction

Cherry tomatoes (Lycopersicon esculentum var. cerasiforme A. Gray) are a popular tomato cultivar rich in lycopene, natural organic acids, essential human amino acids, vitamin C, and various mineral elements. Cherry tomatoes have become an indispensable item on the table with multiple health benefits, including the ability to warm the lung, liver, spleen, and stomach; quench thirst; and prevent cartilage disease and gum bleeding in children [1,2]. Quality indicators such as the soluble solid content (SSC) and pH are crucial for decision making in cherry tomato harvesting, trading, and consumption. However, the current quality judgment of cherry tomatoes primarily depends on the color, size, and shape of the fruits, leading to high labor costs and subjective judgments [3]. 

While using a refractometer for the measurement of the SSC and a pH meter for the measurement of pH can ensure high efficiency and accuracy in single-sample measurements, these methods require destructive sampling and cannot suffice large-scale measurement needs. Hyperspectral imaging technology comprises two-dimensional imaging and one-dimensional spectral technology, with several advantages such as a high resolution, multi-band imaging, and the efficient integration of images. It has been proven as a valuable tool for advanced non-destructive testing in fields like plant disease detection, industrial food packaging, medical imaging classification, horticultural products [4,5,6]. It provides reliable, non-invasive, and quick analyses of essential nutrients like soluble solids, water content, and total acid in the quality detection of fruits [7,8,9,10]. 

Currently, modeling methods for fruit quality attribute prediction involve pre-processing wavelength-screening techniques, such as competitive adaptive reweighted sampling (CARS) and the successive projections algorithm (SPA) to enhance the features while reducing the dimension, and machine learning approaches such as partial least squares (PLS) and the support vector regression (SVR) algorithm are employed for modeling [11,12,13]. Rahman investigated the use of hyperspectral imaging to assess chemical components such as the moisture content (MC), pH, and SSC in intact tomatoes [14]. By developing multivariate corrected models for different pre-processed spectra using PLS regression, the study established that the regression model based on the first-order derivative of Savitzky–Golay (S-G) performed better for MC, pH, and SSC, with correlation coefficients of 0.81, 0.69, and 0.74, respectively. The root mean square errors of prediction were 0.63%, 0.06%, and 0.33%, respectively. Similarly, Sun explored the rapid non-destructive detection of cherry tomato sugar content in the field using the portable near-infrared spectroscopy instrument AMBER II [15]. The uninformative variable elimination–successive projections algorithm (UVE-SPA) was used to extract 12 characteristic wavelength points for PLS modeling, resulting in a coefficient of determination (R^2^) of 0.9385 and 0.9347 for the training set and prediction set, respectively. The application of hyperspectral imaging (HSI) in the detection of tomato quality indicators can be divided into two categories: (1) For the detection of qualitative indicators (damage identification, spoilage detection, and maturity detection), Cho et al. [16] established the detection model of tomato maturity using the support vector machine (SVM) algorithm, and the classification accuracy reached more than 75.00%. (2) For the quantitative detection of the quality ingredients using HIS, Li Liu et al. [17] predicted the SSC of cherry tomatoes using PLS and LS-SVM based on HIS; Anisur Rahman et al. [14] determined the SSC, MC, and pH of cherry tomatoes using PLS based on HSI.

Deep learning enables models to automatically identify features through a vast range of sample data. It is widely used in various industries, including medicine, agriculture, object detection, image recognition, signal processing, and many other fields. By leveraging deep learning regression models and hyperspectral images, Xiang created non-destructive testing techniques for both SSC and fruit hardness [18]. Their findings highlight a new one-dimensional convolutional ResNet-based regression model. When compared to existing regression techniques, the proposed method obtained superior results that showed a 26.4% increase for SSC predictions and a 33.7% increase for hardness, particularly with larger sample sizes. Zhang utilized near-infrared hyperspectral imaging (NIR-HSI) to determine the total phenols, total flavonoids, and total anthocyanins present in black goji berries [19]. Furthermore, they developed a convolutional neural network (CNN) to predict the chemical composition, which yielded comparable performance to conventional methods such as PLS and the least-squares support vector machine (LS-SVM).

At present, there are still some technical bottlenecks to be overcome in the non-destructive testing of tomatoes using hyperspectral imaging technology, such as the improvement of spectral analysis algorithms, the upgrading of instruments and equipment, the control of detection costs, etc. The research and development of sensor hardware is relatively slow. Therefore, the current research of hyperspectral imaging technology in the field of tomato quality detection mainly focuses on data mining in order to improve the accuracy and efficiency of tomato quality detection. Classical modeling methods have the advantage of being easily comprehensible and implementable, and are known to yield positive results with small datasets. Nevertheless, they also exhibit certain disadvantages. For one, it necessitates human intervention to select features and algorithms, requiring operators with high levels of experience and expertise. Furthermore, it relies on the selection and processing of features, which can potentially impact the stability and reliability of modeling outcomes. Deep learning approaches possess end-to-end modeling capabilities that can automatically learn features and patterns from raw data, thereby yielding effective classification and regression outcomes. This technique displays exceptional adaptability to data and generalization abilities, enabling it to handle very large datasets and highly nonlinear problems. In addition, deep learning models are characterized by their high scalability and flexibility, and can effectively process diverse types of data, including images, sound, text, and time-series data. In 2017, the Transformer was introduced as a neural network model capable of processing sequence data exclusively through the use of the attention mechanism [20]. Originally designed for natural language processing (NLP) tasks, Transformers have demonstrated remarkable flexibility and performance, gaining popularity in various applications, such as computer vision (CV) and audio processing. With the advantages in vision tasks, video processing, and music generation, the Transformer framework implemented in Vision Transformer (ViT) and BERT facilitates a performance enhancement of their models specifically tailored to the task at hand. Therefore, this study presented a Transformer-based regression model to predict the SSC and pH of cherry tomatoes using hyperspectral data. Although CNN approaches have been widely used for spectra data analysis due to their ability to learn deep features [19], the Transformer, as a different deep learning approach with a strong ability to learn deep features, has not been used for regression with spectra.

This study employed a combination of hyperspectral imaging techniques and deep learning-based regression models to predict the SSC and pH of cherry tomatoes, and verified the self-attention mechanism of the Transformer for regression using spectra data for the first time. The combination of a CNN and the Transformer was also explored to enhance the feature-learning ability of the two deep learning approaches. This study is guided by three specific aims: (1) to establish traditional regression models (PLSR, SVR) and deep learning regression models (CNN, LSTM, and Transformer) for SSC and pH prediction; (2) to improve the CNN-Transformer model regression effect; and (3) to promote model interpretability between diverse wavelengths and the SSC and pH.

## 2. Materials and Methods

### 2.1. Sample Preparation

In mid-July 2022, a total of 357 cherry tomatoes (cv. ZheyingFen 1) were collected in five batches from a greenhouse located in Changxing County, Huzhou, Zhejiang Province, China. These samples comprised 177 ripe tomatoes, with over 90% displaying a red fruit color, and 180 immature tomatoes, with over 70% displaying a red fruit color. In order to control the experimental variables, all samples were intact and the fruit surface was clean. The fruit pedicle was uniformly removed for data collection. Hyperspectral image acquisition and reference value measurement of the SSC and pH were accomplished on the day of sample collection of each batch of samples. 

### 2.2. SSC and pH Measurements

The SSC content was tested using a sugar refractometer (BR0035, WIGGENS Technology Ltd., Beijing, China), while the pH was determined using a Sartorius pH meter (PB-10, Sartorius, Gottingen, Germany). Upon the completion of hyperspectral image acquisition, a solitary cherry tomato sample was juiced via manual juicing and subsequently filtered with gauze. The SSC content was ascertained by dispensing 1 mL of juice into the refractometer, whilst the pH was measured through the use of the pH meter probe within the juice. To prevent any potential contamination, the refractometer, pH meter probe, and juicer were carefully washed with distilled water after each measurement and wiped clean with paper towels prior to the next reading. Expressed in Table 1, the resultant sample measurements are featured.

### 2.3. Hyperspectral Imaging System

**Figure 1:**
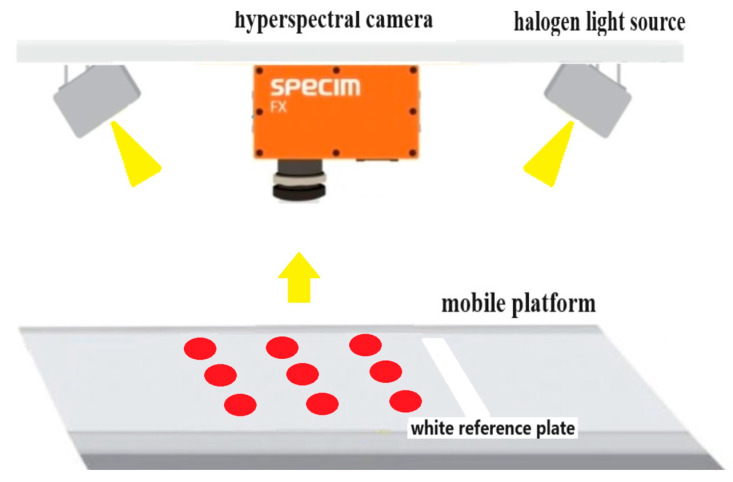
Hyperspectral imaging system.

In this study, hyperspectral images of cherry tomatoes were collected using a hyperspectral imaging system. The hyperspectral camera was FX-17 (Spectral Imaging Ltd., Oulu, Finland), the spectral range was 900–1700 nm, and the number of spectral bands was 224. The spectral resolution was 8 nm. Two groups of halogen light sources were symmetrically located on both sides of the hyperspectral camera for illumination, and each group of light sources consisted of three halogen bulbs. Each halogen bulb has a power of 35 w. During image acquisition, the moving speed of the mobile platform was set to 25 mm/s, the exposure time was set to 8.26 ms, and the hyperspectral image was obtained through push-scan imaging (line scanning) from left to right, as shown in Figure 1. The cherry tomatoes were evenly placed on the platform in 3 rows and 5 columns in a single capture process. Then, a white reference plate was placed on the front end to obtain the white reference image for light intensity calibration, and a black opaque lens cover with the lamp off was used to obtain the dark reference image [21]. The corrected image could be obtained by applying Formula (1). An RGB image and the grey-scale images at 1008, 1203, and 1403 nm of one hyperspectral image are shown in Figure S1 (in the Supplementary Materials).

(1) $$R=\frac{I-D}{W-D}$$

where *I* is the original image, *R* is the corrected image, *W* is the white reference image, and *D* is the black reference image.

### 2.4. Spectra Extraction and Processing

**Figure 2:**
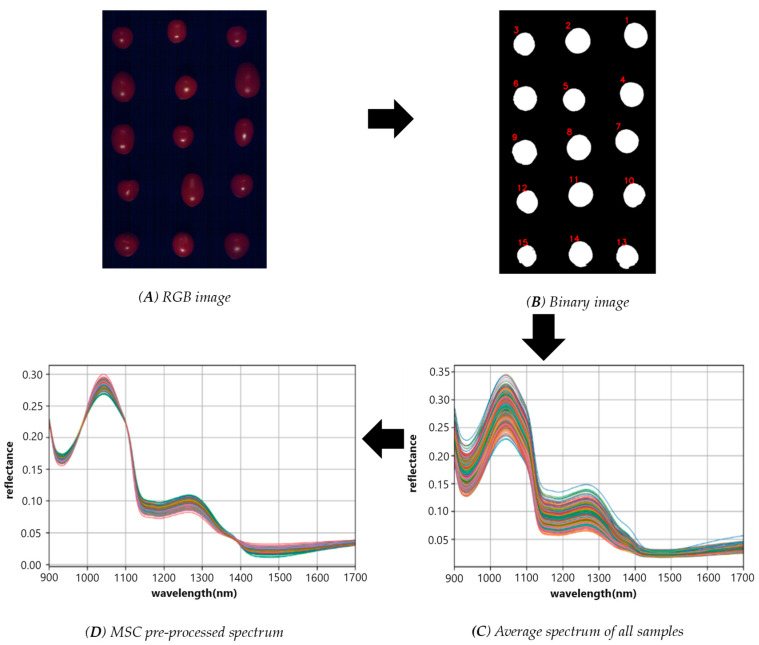
(**A**) RGB image; (**B**) the binary image of the cherry fruit region; (**C**) the spectra of all samples obtained by calculating the average of all the pixels within the same sample; and (**D**) the MSC pre-processed spectrum.

The entire region of each tomato in the hyperspectral images was defined as the region of interest (ROI). The ROIs were extracted using OpenCV image processing, and the average spectrum of all pixel directional spectra within the ROI was obtained to represent the spectral profile of the tomato. To ensure accurate detection results, the noisy ends of the original spectral curve were eliminated by selecting the spectrum with smoother information. Box plots were employed to eliminate outliers, and multiplicative scatter correction (MSC) was utilized to pre-process the spectra. The process of spectrum extraction and processing is depicted in Figure 2. 

### 2.5. Partitioning the Sample Set

To establish and compare the predictive effects of different models, the samples were randomly divided into the calibration set, the validation set, and the prediction set at a ratio of 3:1:1. The calibration set was used to train the model, while the validation set was utilized to assess the model’s robustness. The prediction set was used to evaluate the generalization capability of the final model. Since improper random data partitioning might result in regression overfitting, PLSR regression was employed to evaluate the performances of a random split of datasets. After trials, the datasets corresponding to the PLSR which had good prediction results and was free from overfitting were obtained and used for further analysis.

### 2.6. Regression Models

#### 2.6.1. PLSR

Partial least squares regression (PLSR) [22] tackles the modeling problem involving an independent variable set (X) and dependent variable set (Y). Initially, the first pair of principal components (t and u) are derived from X and Y, respectively, with the aim of maximizing their correlation degree. This process of extracting principal components is repeated until the regression equation reaches an acceptable level of accuracy. Ultimately, this approach can serve as a useful linear method in studying both spectrum and reference values. For this particular research, the number of principal components to be tuned was utilized in the range of 1–21. The optimal number of principal components was determined through the use of an automated parameter adjustment technique, known as GridSearchCV (function of model_selection in sklearn, grid search to find the global optimal value with cross-validation). 

#### 2.6.2. SVR

SVR is an application of support vector machines (SVMs) that presents unique advantages in addressing small-sample, nonlinear, and high-dimensional pattern recognition challenges [23,24]. In linear cases, an SVM constructs classifiers by maximizing the distance between different classifications and learning linear functions to classify samples. In nonlinear cases, the SVM transforms low-dimensional features into high-dimensional feature spaces via kernel functions, causing problems to become linear in that space. Ultimately, an optimal classification hyperplane is established in the feature space, and the SVM model produces an optimal solution. This study uses various kernel functions such as linear, polynomial, and Gaussian, combined with a cross-verified mesh search to optimize parameter gamma in the range of [10^−4^, 10^5^].

#### 2.6.3. CNN

The CNN serves as one of the representative algorithms of deep learning and utilizes convolutional check variables to extract features. The convolution kernel parameter sharing in the hidden layer combined with the sparsity of inter-layer connections enable the CNN to efficiently lattice features with minimal computation, making it a useful tool in computer vision, natural language processing, and other fields [21]. In the current study, the proposed CNN model included two one-dimensional convolution layers, utilizing 128 and 256 channels, respectively, with each convolution kernel size set to 1 × 3. To reduce the influence of gradient disappearance and improve the calculation speed, the activation function was selected as “ReLU”. The dropout layer was employed after the fully connected layer, and the probability was set to 0.3 to prevent the overfitting of the model. To expedite the convergence rate, the learning rate was set to 0.0005, the batch size was set to 10, and the AdamW optimizer was employed during training.

#### 2.6.4. Long Short-Term Memory (LSTM) and CNN-LSTM

The LSTM network is a specialized RNN model that addresses long-term dependency issues through a unique structural design. Unlike other models that require expensive measures to retain early information, the LSTM’s default behavior is to store such data. This is achieved through the integration of three distinct gates: forgetting, memory, and output gates. These gates are responsible for managing the retention and transmission of information within the LSTM, as detailed in the literature [25]. In this study, a linear transformation was utilized to pre-process data before inputting a one-way LSTM model with 1024 input dimensions and 1024 hidden-layer dimensions.

Additionally, for the CNN-LSTM model in this study, CNN feature extraction was implemented before inputting the LSTM, and three-layer, one-dimensional convolution and a 3 × 3 convolution kernel were adopted. The number of channels in the convolution kernels was 128, 256, and 512, respectively. The one-way LSTM with 512 inputs and 256 hidden dimensions was used for the CNN-LSTM.

#### 2.6.5. Transformer and CNN-Transformer

The Transformer system comprises two fundamental components—an encoder and a decoder. The Multi-Head-Attention in the encoder uses multiple heads to form multiple subspaces, which allows the model to focus on different aspects of information and learn complex understanding requirements in the context through the layer stacking of attention. In this study, only the encoder component of the Transformer was utilized to isolate the attention information, finally feeding it to the full connection layer regression output [20,26,27,28]. The Multi-Head-Attention function of the system can be defined as a process that maps a query and a set of key–value pairs to produce an output, where all components, namely the query, key, value, and output, are represented as vectors. The output is obtained by calculating a weighted sum of the provided values, where each weight is derived from the compatibility function between the query and its corresponding key. The multi-head enables the system to efficiently concentrate on diverse representation subspaces across multiple locations. The use of an attentive head can help to overcome the potential of averaging that may result in the suppression of useful information.

**Figure 3:**
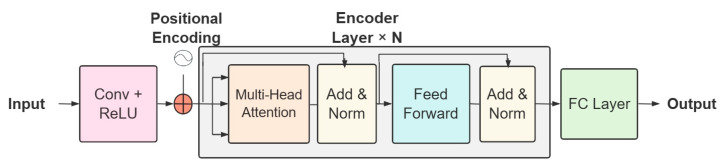
CNN-Transformer network structure schematic.

The CNN-Transformer model diagram is depicted in Figure 3. The initial step involved feeding the input spectral data through the convolutional layer to extract features, with the number of convolutional cores being 128 and 256 after two convolutional operations. For positional encoding, positional functions were utilized to enable the model to concentrate on relative positional information, as shown in Formula (2). The encoder layer, based on the debugging outcome, comprised 6 layers, and each layer was constructed with Multi-Head-Attention, Feed Forward, and Add and Normalize. The residual standardized connection consisted of Add and Norm, with its computation depicted in Formula (3). Multi-Head-Attention was the essence of the Transformer, and four heads were employed to form multiple subspaces that enable the model to concentrate on different aspects of the information, estimating the correlation between different spectral bands and the output residual connection of the convolutional layer, as shown in Formula (4). Finally, the predicted results were obtained by the fully connected neural network (FC layer).

(2) $$\begin{array}{c} \mathrm{PE}(pos,2i)=\sin\left(\frac{\;\mathrm{pos}\;}{10000^{2i/d_{\mathrm{model}\;}}}\right)\\ \mathrm{PE}(\mathrm{pos},2i+1)=\cos\left(\frac{\;\mathrm{pos}\;}{10000^{2i/d_{\mathrm{model}\;}}}\right) \end{array}$$

where PE indicates the position code, pos indicates the position of the current character in the input letter, and $d_{\mathrm{model}}$ is the representation dimension.

LayerNorm (*X* + MultiHeadAttention (*X*))

LayerNorm (*X* + FeedForward (*X*)) (3)

(4) $$Attention(Q,K,V)=softmax\left(\frac{QK^{T}}{\sqrt{d_{k}}}\right)V$$

where *Q*, *K*, and *V* are all obtained through the transformation of input spectral variables, and *d_k_* represents the variable dimension.

When training the CNN-Transformer model, the hyperparameters of the model were set, with a learning rate of 0.0008, a batch size of 10, a source sentence length of 209, a target length of 1, a word vector dimension of 256, a hidden layer in the twice linear layer of 1024, and a *K* = *Q* = *V* dimension of 64. The number of encoder layers was set to 6; the number of headers was set to 4. MSELoss was used as the loss function, and the AdamW optimizer was used for training to speed up the convergence.

### 2.7. Software, Hardware, and Performance Evaluation

All models were constructed using Python (version 3.6), with the deep learning framework PyTorch (version 1.9.0) utilized to establish the model’s architecture and execute calculations on the GPU. The SVR and PLSR were modeled using Scikit-learn (version 0.24.2). All data analysis was carried out on a computer containing an Intel (R) Core (TM) i5-10500H processor, operating at 2.50 GHz, and 16 GB of RAM, alongside an NVIDIA GeForce RTX 3050 Laptop GPU.

To compare the performances of the PLSR, SVR, CNN, LSTM and Transformer models, the root mean square error (RMSE) (utilized as Formula (5)), the coefficient of determination (R^2^) (utilized as Formula (6)), and the relative percentage difference (RPD) (utilized as Formula (7)) were used as performance evaluation indicators.

(5) $$RMSE=\sqrt{\frac{1}{m}\sum_{i=1}^{m}{\left(y_{i}-{\hat{y}}_{i}\right)}^{2}}$$

(6) $$R^{2}=1-\frac{\sum_{i}{\left({\hat{y}}_{i}-y_{i}\right)}^{2}}{\sum_{i}{\left({\bar{y}}_{i}-y_{i}\right)}^{2}}$$

$y_{i}$: true value; ${\hat{y}}_{i}$: predicted value; ${\bar{y}}_{i}$: mean of true value.

(7) $$RPD=\frac{SD}{SEP}$$

*SD*: the standard deviation; *SEP*: the root mean square error 

The R^2^ provides insight into the ability of the independent variable (x) to account for changes in the dependent variable (y). A higher R^2^ value indicates a better fit for the model. Likewise, the RMSE gauges the mean square variance between predicted and actual result values for the model. A lower RMSE is indicative of a better model performance. RPD < 1.4 means that the model is not reliable, 1.4 < RPD < 2.0 means that the model is considered reliable, and RPD > 2.0 means that the built model has high reliability and can be used for model analysis.

## 3. Results and Discussion

### 3.1. Spectral Profiles

In this study, 357 cherry tomato samples were analyzed and studied. The wavelength range of the full spectrum data was 900–1700 nm, and the noise at both ends of the original spectral curve was relatively large. The spectral bands between 900 and 945 nm were removed from the beginning, and only the spectra in the range of 946–1700 nm were used for further analysis. MSC was used for spectral correction. The principle is to conduct unary linear regression between the spectrum of each sample and the average spectrum, find the baseline translation and offset of each sample, and then correct the spectrum to eliminate the spectral difference caused by different scattering levels. Figure 2C shows the spectral data after MSC treatment; subtle differences can be observed in different samples, and two distinct peaks can be observed at 1000–1100 nm and 1220–1300 nm. The relationship between the spectrum and the SSC and pH was further discussed by means of machine learning and deep learning models.

### 3.2. Regression Model Establishment

To establish the regression models, the full-range spectra were used. The conventional PLSR and SVR methods were used to develop regression models using full-range spectra. Impressively, promising outcomes are observed and evidenced in Table 2. With regard to the SSC estimation, both models attained $R_{c}^{2}$, $R_{v}^{2},$ and $R_{p}^{2}$ values above 0.8; both models attained RPDP values above 2.4. However, the PLSR model outperformed the SVR model in the pH prediction domain, with $R_{c}^{2}$, $R_{v}^{2},$ and $R_{p}^{2}$ values of approximately 0.6. PLSR obtained an RPDP value of 1.82, which was the best performance of all models on the prediction set. Anisur Rahman et al. used the spectra in the range of 1000–1550 nm, and after MSC treatment, established a PLS model to predict the SSC and pH, with an Rpred of 0.78 and 0.32, respectively [14]. Hence, it could be deduced from these findings that the combination of hyperspectral imaging with traditional regression methods showed an excellent outcome in SSC prediction, but its potential in pH prediction remains unveiled.

**Figure 4:**
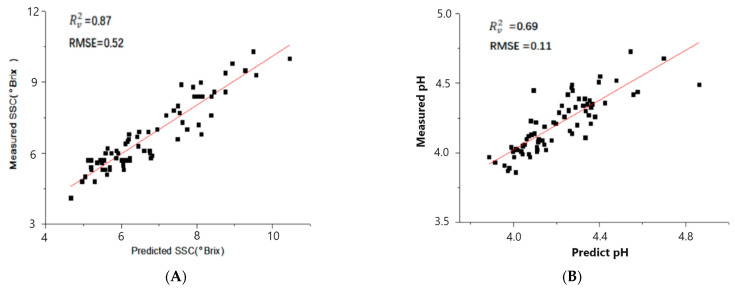
CNN-Transformer scatter diagrams. (**A**) Scatter diagram for SSC prediction validation set; (**B**) scatter diagram of pH prediction validation set.

Additionally, deep learning methods including CNN, Transformer, and LSTM were used for regression analysis. Using the feature extraction ability of the CNN, the model parameters were adjusted to build the CNN-LSTM and CNN-Transformer models. In the prediction of the SSC, the $R_{c}^{2}$, $R_{v}^{2},$ and $R_{p}^{2}$ values of the CNN and LSTM models were found to be between 0.5 and 0.6, and both models attained RPDP values above 1.5. The Transformer model performed relatively better, with an R^2^ value around 0.7 (Table 2), and the RPDP value was 1.88. By incorporating spectral data features extracted by the CNN into the Transformer encoder, attention could be weighted to produce a more accurate prediction. It could be observed that the performance of the CNN-LSTM models were slightly improved compared to LSTM. The CNN-Transformer showed a particularly significant improvement compared to the Transformer, achieving $R_{c}^{2}$, $R_{v}^{2},$ and $R_{p}^{2}$ values of over 0.83 in SSC prediction. The scatter diagrams of the predicted and measured values of cherry tomatoes based on the CNN-Transformer model is shown in the Figure 4. The red line is the regression line representing the ideal correlation between the predicted value and the measured value, and the closer to the regression line, the better the model prediction is shown. It is worth mentioning the RPDP value of 2.45, which was the best performance of all models on the prediction set. In the prediction of pH, an R^2^ near 0.69 and an RPDP near 1.6 (Figure 4) were obtained. In a similar study, Yun Xiang et al. used deep learning and hyperspectral images to predict the SSC and firmness of tomatoes. In terms of the SSC, the R^2^ of the deep learning model was 0.49 in a small sample set (50 samples). Notably, the R^2^ was 0.9 in a large sample (200 samples) of the deep learning model, and the result was better than PLSR, AdaBoost, KNNR, and SVR [18].

In comparison, it was observed that the performances of SSC prediction exceeded the performances of pH prediction. However, it was worth emphasizing that the optimization of the deep learning model’s structure could greatly improve its predictive capabilities. This was particularly true for the CNN-Transformer model, which exhibited remarkable performance in accurately predicting both the SSC and pH. Notably, the results showed that the SSC prediction accuracy obtained by the CNN-Transformer was comparable to that obtained by traditional methods such as PLSR and SVR. Moreover, it showcased excellent performance in predicting pH values. Empirical evidence unequivocally indicated the efficacy of the CNN-Transformer model in achieving precise and timely cherry tomato internal quality prediction. Additionally, the flexible structure of this model presents ample opportunities for further refinement and advancement, thereby augmenting its overall potential.

### 3.3. CNN-Transformer Network Visualization

The interpretability of the CNN-Transformer model was investigated by analyzing the weight distribution corresponding to different wavelengths in the prediction of the SSC and pH, respectively. Through the use of Grad-CAM, gradient information was extracted from the specified feature layer via backpropagation. This information was then utilized to calculate the importance of each channel within the feature layer, resulting in a heat map that allowed for an analysis of the model’s acquisition of relevant feature information. While Grad-CAM was commonly employed for visualizing image classification problems, it could also be adapted to regression problems by utilizing gradient computation to capture the significance of input variables.

**Figure 5:**
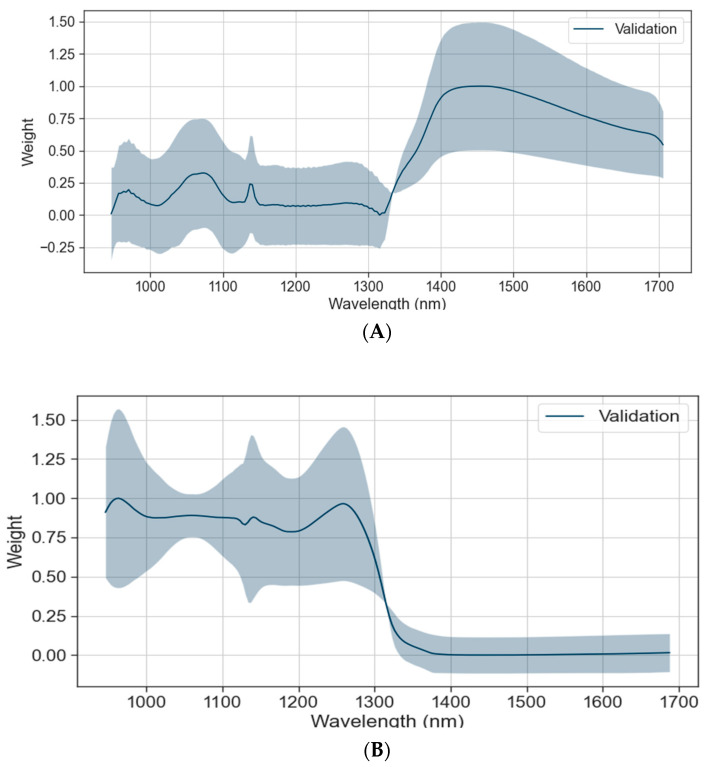
(**A**) Weight distribution corresponding to wavelength of CNN-Transformer calculated by Grad-CAM for SSC; (**B**) weight distribution corresponding to wavelength of CNN-Transformer calculated by Grad-CAM for pH.

To explore interpretability, a randomly selected set of 50 samples was inputted into the pre-trained model. Grad-CAM was subsequently applied to extract gradient information from the final convolutional layer’s feature map. The resulting weight vector and the associated channel of the feature map were linearly weighted and averaged to generate a one-dimensional weight distribution map. The weights corresponding to different wavelengths are shown in the Figure 5, where the blue shadow represents the variance of the samples, and the larger the shadow area, the larger the variance, indicating the greater the dispersion from the mean value.

#### 3.3.1. Important Wavelengths for SSC Prediction

The Grad-CAM analysis showed that in the prediction of the SSC in the CNN-Transformer (Figure 5A), the weights of wavelengths in the range of 1380–1650 nm were the highest. The 1380 nm region was attributed to the influence of hydroxide in water, and it is speculated that the nearby wavelength is the main factor affecting the SSC. The weights of other wavelengths were relatively low, which also had an impact on the prediction performances.

Liu tested the sugar content of cherry tomatoes based on HSI [17,29]. Spectral curves between the wavelengths of 914.91 nm and 1661.91 nm were used to extract feature bands by CARS, SPA, and SPA-CARS. There were 36 wavelengths extracted by CARS, and there were 10 characteristic bands in the spectral range of 1380–1650 nm. There were 40 wavelengths extracted by SPA, and there were 24 characteristic bands in the spectral range of 1380–1650 nm. There were 35 wavelengths extracted by SPA, and there were 20 characteristic bands in the spectral range of 1380–1650 nm. The results were similar to the conclusion of this study. Li studied the online quality detection system of cherry tomatoes based on short-wave near-infrared spectroscopy. Nineteen variables were extracted from 900 to 1700 nm by CARS-PLS, nine variables were distributed from 900 to 1200 nm, and 10 variables were distributed from 1400 to 1700 nm, which was similar to the conclusion of this study [30,31]. The experimental results showed that the characteristic wavelength range of 1380–1650 nm was the most important factor in predicting the SSC of cherry tomatoes, followed by the characteristic wavelength range of 900–1100 nm.

#### 3.3.2. Important Wavelengths for pH Prediction

Based on the analysis conducted, it was shown that the wavelength range of 945–1280 nm held the highest significance in predicting the pH levels in the CNN-Transformer (Figure 5B). Li used the SPA and PLSR to predict the SSC and pH of cherry tomatoes in the 874–1734 nm region, and the distribution of pH characteristic bands in the spectral curve was highly consistent with the experimental results [9]. The range of wavelengths from 972 nm to 1200 nm appeared to have a significant impact on the prediction results. This prominence in prediction could be attributed to the vibration of hydrogen groups, especially the O-H and N-H groups. These hydrogen groups were commonly found in acidic compounds and were known to influence pH predictions.

### 3.4. Discussion

This study examined the impact of traditional methods and deep learning on predicting SSC and pH indicators of cherry tomatoes, with a focus on the Transformer model and CNN-Transformer model. The PLSR, SVR, and CNN-Transformer models all demonstrated excellent predictive performances for the SSC, with R^2^_C_, R^2^_V_, and R^2^_P_ values all exceeding 0.8. PLSR and the CNN-Transformer also performed well in predicting pH, with R^2^_C_, R^2^_V_, and R^2^_P_ values all exceeding 0.6. Research indicates that traditional methods such as PLSR and SVR remain reliable. However, by combining the extraction features of the CNN with the self-attention mechanism of the Transformer, it could be observed that the enhanced CNN-Transformer model has made significant progress in predicting SSC and pH in cherry tomatoes. This study observes that deep learning surpasses traditional machine learning algorithms in terms of better generalization across various metrics and adaptability to different datasets. Furthermore, in the context of predicting the levels of dry black goji berries, as well as flavonoids and anthocyanins in black wolfberry, the utilization of convolutional neural networks (CNNs) as a modeling and feature extraction method demonstrated favorable and comparable results when compared to the conventional approach [32]. The success of deep learning techniques indicates their promising potential as a valuable method for prediction and feature extraction in relevant fields. 

The prediction effect of the pH of all models in this experiment was worse than that of the SSC, and the same conclusion could be found in other studies. Anisur Rahman et al. developed a multivariable calibration model using hyperspectral imaging and PLSR for pH and SSC, and the predictive effect of the pH was inferior to that of the SSC, with correlation coefficients of prediction (r_pred_) of 0.75, 0.69, and 0.74 [14]. Yuping Huang et al. developed spatially resolved spectroscopy (SRS) to assess tomato quality. The prediction effect of the pH was lower than that of the SSC [33]. Hongbin Pu et al. employed hyperspectral imaging to forecast the sugar content and acidity of litchi. It could be found that the prediction of pH was worse than the prediction of SSC [34].

Grad-CAM was utilized for extracting gradient information from the specified feature layer through backpropagation to analyze the weight distribution corresponding to different wavelengths when predicting SSC and pH in the CNN-Transformer model. It was observed that the weight for predicting the SSC and pH differed significantly. The wavelength range of 1380–1650 nm was the key region for predicting the SSC, while the wavelength range of 945–1280 nm was the key region for predicting the pH. These findings align with those of other papers and demonstrate that the CNN-Transformer model could effectively learn spectral information for reliable prediction [9,17,29,31].

## 4. Conclusions

In this study, multiple predictive regression models such as PLSR, SVR, CNN, LSTM, CNN-LSTM, Transformer, and CNN-Transformer were developed to perform non-destructive testing of the SCC and pH of cherry tomatoes. The results showed that traditional machine learning still has a stable performance in predicting SSC, but the effect of predicting pH is not so good. Deep learning methods have experienced great changes, among which the CNN-Transformer model has an excellent effect in predicting the SSC and pH value of cherry tomatoes; the R^2^ values were around 0.87 and 0.7, respectively. The characteristic wavelength distribution of the CNN-Transformer model was visualized using Grad-CAM, which enhances the interpretability of the model. In conclusion, the CNN-Transformer could enhance the accuracy of predicting the SSC and pH of cherry tomatoes using hyperspectral imaging. It offers an efficient method for the non-destructive testing of the tomato quality index and other types of fruits. The application of hyperspectral imaging technology with deep learning in fruit quality detection can improve the quality and value of fruit. It can also drive and promote the development of the entire fruit industry chain and improve the competitiveness of the fruit industry by using advanced detection techniques and artificial intelligence.

## Figures and Tables

**Table 1:** Results of sample SSC and pH measurements.

Number of Samples	SSC (°Brix)				pH			
	Mean	Maximum	Minimum	Standard Deviation	Mean	Maximum	Minimum	Standard Deviation
357	6.90	10.20	4.20	1.41	4.20	4.78	3.77	0.20

**Table 2:** Results of PLSR, SVR, CNN, LSTM, Transformer, CNN-LSTM, and CNN-Transformer models for SSC and pH prediction.

Indicators	Models	Calibration		Validation		Prediction		
		R^2^_C_	RMSEC	R^2^_V_	RMSEV	R^2^_P_	RMSEP	RPDP *
SSC	PLSR	0.80	0.55	0.82	0.58	0.84	0.56	2.44
	SVR	0.90	0.39	0.87	0.52	0.85	0.59	2.34
	CNN	0.50	0.95	0.68	0.81	0.50	0.97	1.41
	LSTM	0.56	0.89	0.54	0.98	0.56	0.91	1.52
	Transformer	0.62	0.82	0.73	0.75	0.72	0.73	1.88
	CNN-LSTM	0.57	0.88	0.55	0.98	0.56	0.91	1.50
	CNN-Transformer	0.83	0.58	0.87	0.52	0.83	0.56	2.45
pH	PLSR	0.66	0.10	0.61	0.12	0.73	0.10	1.82
	SVR	0.75	0.08	0.55	0.13	0.58	0.13	1.53
	CNN	0.60	0.12	0.58	0.12	0.54	0.13	1.48
	LSTM	0.47	0.14	0.42	0.15	0.48	0.14	1.39
	Transformer	0.51	0.14	0.56	0.13	0.63	0.12	1.64
	CNN-LSTM	0.73	0.10	0.53	0.13	0.61	0.12	1.60
	CNN-Transformer	0.74	0.10	0.69	0.11	0.60	0.12	1.59

* RPDP: The difference in precision of RMSE results in the difference in RPD.

## Data Availability

The data can be requested by contacting the corresponding authors via E-mail.

## Supplementary Materials

The following supporting information can be downloaded at https://www.mdpi.com/article/10.3390/foods13020251/s1: Figure S1: (a) RGB image of cherry tomato; (b1) corrected grayscale image at 1008 nm; (b2) corrected grayscale image at 1203 nm; (b3) corrected grayscale image at 1403 nm.
