# Supplementary material for: Hyperspectral Imaging Using a Convolutional Neural Network with Transformer for the Soluble Solid Content and pH Prediction of Cherry Tomatoes
Source: Foods. 2024 Jan 12;13(2):251. doi: 10.3390/foods13020251 (PMC10814136; doi:10.3390/foods13020251)
Supplement: Supplementary file 1 [file foods-13-00251-s001.zip › foods-2751902-supplementary.pdf]

Supplementary Materials

Hyperspectral Imaging Using a Convolutional Neural Network with Transformer for the Soluble Solid Content and pH Prediction of Cherry Tomatoes

Hengnian Qi [1,†], Hongyang Li [1,†], Liping Chen [2,*], Fengnong Chen [3], Jiahao Luo [1] and Chu Zhang [1,*]

[1] School of Information Engineering, Huzhou University, Huzhou 313000, China
[2] Huzhou Agricultural Science and Technology Development Center, Huzhou 313000, China
[3] School of Artificial Intelligence, Hangzhou Dianzi University, Hangzhou 310018, China
* Correspondence: chenliping20042000@163.com (L.C.); chuzh@zjhu.edu.cn (C.Z.)
† These authors contributed equally to this work.

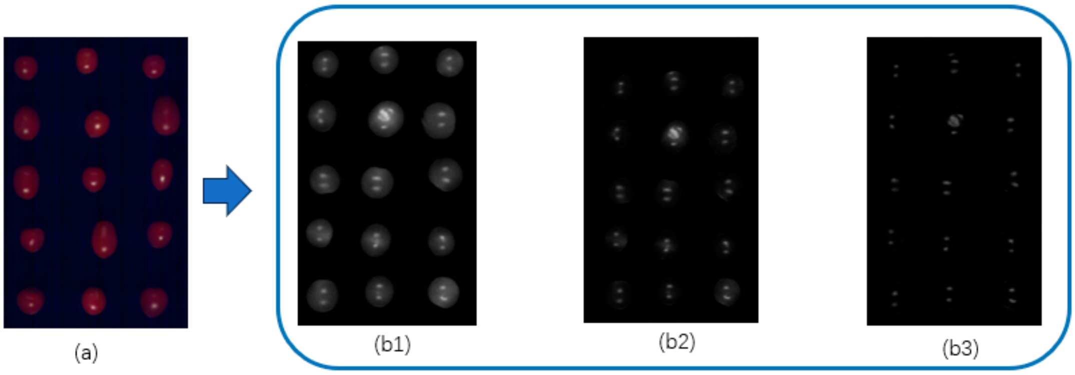

Figure S1. (a) RGB image of cherry tomato; (b1) Corrected grayscale image at 1008 nm; (b2) Corrected grayscale image at 1203 nm; (b3) Corrected grayscale image at 1403 nm
